# The *NAC* Transcription Factor *PgNAC41-2* Gene Involved in the Regulation of Ginsenoside Biosynthesis in *Panax ginseng*

**DOI:** 10.3390/ijms241511946

**Published:** 2023-07-26

**Authors:** Chang Liu, Mingzhu Zhao, Hedan Ma, Yu Zhang, Qian Liu, Sizhang Liu, Yanfang Wang, Kangyu Wang, Meiping Zhang, Yi Wang

**Affiliations:** 1 College of Life Science, Jilin Agricultural University, Changchun 130118, China; lchang1205@163.com (C.L.); marrco_00@163.com (H.M.); m13906495700@163.com (Y.Z.); m18653220658@163.com (Q.L.); lsz512411412@163.com (S.L.); kangyu.wang@jlau.edu.cn (K.W.); meiping.zhang@jlau.edu.cn (M.Z.); 2 Jilin Engineering Research Center Ginseng Genetic Resources Development and Utilization, Changchun 130118, China; 3 Laboratory for Cultivation and Breeding of Medicinal Plants of National Administration of Traditional Chinese Medicine, Jilin Agricultural University, Changchun 130118, China; yfwang2014@163.com

**Keywords:** *PgNAC41-2* gene, ginsenoside, expression analysis, vector construction, genetic transformation

## Abstract

Ginseng (*Panax ginseng* C.A. Meyer) is a perennial herb of the Araliaceae family, a traditional and valuable Chinese herb in China. The main active component of ginseng is ginsenoside. The *NAC* transcription factors belong to a large family of plant-specific transcription factors, which are involved in growth and development, stress response and secondary metabolism. In this study, we mapped the NAC gene family on 24 pairs of ginseng chromosomes and found numerous gene replications in the genome. The *NAC* gene *PgNAC41-2*, found to be highly related to ginsenoside synthesis, was specifically screened. The phylogeny and expression pattern of the *PgNAC41-2* gene were analyzed, along with the derived protein sequence, and a structure model was generated. Furthermore, the *PgNAC41-2* gene was cloned and overexpressed by a *Rhizobium rhizogenes* mediated method, using ginseng petioles as receptor material. The saponin content of the transformed material was analyzed to verify the function of the NAC transcription factor in ginseng. Our results indicate that the *PgNAC41-2* gene positively regulates the biosynthesis of saponins.

## 1. Introduction

The NAC (NAM, ATAF and CUC2) family of transcription factors is one of the largest families of plant-specific transcription factors [1,2]. The first *NAC* gene cloned was the *NAM* (no apical meristem) gene in *Petunia*, which was shown to be involved in the formation of the apical meristem [3]. Subsequently, it was found that Arabidopsis *ATAF1/2* and *CUC2* had similar functions to that of the *NAM*, and the N-terminus of the proteins encoded by these three genes contained a conserved sequence of 150–160 amino acids, thus named as the NAC domain [4] because they are involved in nuclear localization, DNA binding and formation of homodimers or heterodimers with other proteins containing the NAC domain [5]. The N-terminal, the highly conserved DNA-binding domain of the NAC protein, contains five subdomains (A–E) [6]. The C-terminal region is a highly diverse transcriptional regulatory region that can activate or inhibit a variety of downstream genes, thus participating in multiple cellular or molecular processes. The diversity of the C-terminal sequence is the main reason for the regulatory differences between the transcriptional activation activities of NAC proteins [7,8,9]. In addition, some NAC transcription factors also contain α-helical transmembrane (TM) motifs at their C-terminus, which are responsible for anchoring to the plasma membrane or endoplasmic reticulum. These NAC transcription factors are membrane-associated and thus called NAC membrane-bound transcription factors [10,11]. *NAC* transcription factors are involved in many biological processes with diverse and important functions, and they play important roles in plant secondary wall biosynthesis [12,13], the development of flowers and fruits in plants [14,15], plant senescence [16], resistance to abiotic stress [17] and regulation of secondary metabolism [18].

The rice transcription factor OsNAC6 upregulates the expression of direct target genes involved in membrane modification, nicotianamine (NA) biosynthesis, glutathione relocation, 3′-phophoadenosine 5′-phosphosulphate accumulation and glycosylation, which represent multiple drought tolerance pathways. Moreover, the overexpression of *NICOTIANAMINE SYNTHASE* genes, direct targets of OsNAC6, promotes the accumulation of the metal chelator NA and, consequently, drought tolerance [19]. *BoNAC019*, a homolog of *AtNAC019*, was also isolated from Chinese cabbage. Under drought conditions, the content of antioxidant enzymes and anthocyanins in BONAC019-OE plants decreased, resulting in the accumulation of more reactive oxygen species (ROS) and further damage to Arabidopsis [20]. The downregulation of *AeNAC83* caused by virus-induced gene silencing enhanced plant sensitivity to salt stress and increased the biomass accumulation of okra seedlings; meanwhile, *AeNAC83OE* Arabidopsis lines displayed an improved salt tolerance and exhibited several altered phenotypes, including a small rosette, short primary roots and promoted crown roots and root hairs [21]. In the process of papaya fruit ripening, transcription factor *CpNAC1* plays a positive regulatory role in carotenoid biosynthesis by activating the expression of *CP-PDS2/4* [22]. The *MpNAC52* transcription factor in apple (*Malus pumila*) binds to promoters of *MpMYB9* and *MpMYB11*, stimulates the biosynthesis of anthocyanins and proanthocyanins (PA) and also affects the metabolism of PA by regulating colorless anthocyanin reductase [23]. *PdWND3A* in *Populus deltoides* is a member of the protein family containing the NAC domain and a homologous protein of ATVND4/5 in *Arabidopsis thaliana*, which regulates lignin biosynthesis by regulating the expression of the *F5H* gene [24]. Lv et al. [18] studied *NAC* transcription factors in *Artemisia annua* and found that when *AaNAC1* was overexpressed, the content of artemisinin and dihydroartemisinic acid increased by 79% and 150%, respectively, and the expression level of artemisinin biosynthesis pathway genes increased. Dalman et al. showed that the expression of three key genes in the flavonoid biosynthesis pathway, *CHS*, *F3’H* and *PaLAR3*, was always downregulated in Norwegian spruce lines overexpressing *PaNAC03*, indicating a decrease in the level of specific flavonoids [25].

*Panax ginseng* (*Panax ginseng* C.A. Meyer) is a perennial herb of the genus *Panax ginseng* in the family Araliaceae. It was named by the Russian scholar Carl. Anton. Meyer in 1843 [26]. As a precious Chinese medicinal material, ginseng has been used in China for more than 1700 years. It is recorded in *Shennong Materia Medica* that ginseng has the functions of filling the five viscera, stopping palpitation, restating the soul, keeping the spirit safe, removing evil spirits, brightening the eyes, making people happy, and keeping the body light for a long time, which has high medical and health value [27]. Ginsenosides exist in the roots, stems, leaves, flowers, fruits and other parts of ginseng [28]. Ginsenosides can be divided into pentacyclic triterpenoid saponins (oleanolic acid saponins) and tetracyclic triterpenoid saponins (damarane saponins) according to their basic skeleton [29]. According to the different ginsenosides of aglycones, they can be divided into 20 (S)-proto-ginsenodiols, ginsenosides -RB1, -RB2, -RB3, -RC, -RD, etc., 20 (S)-progenitor ginsentriols, ginsenosides -RE, -RF, -RG1, -RG2, -RH1, etc., and oleanolic acids, such as ginsenoside-RO [30,31]. At present, the main active saponin ingredients of ginseng are of interest for medicine and research and development. Some obvious shortages in the supply become evident. In order to meet the market demands, but also to protect the limited resources of wild ginseng and the stability of its ecological environment, an application of modern biology technology and the use of innovative germplasm collections aiming to increase the production of ginseng saponin is in the focus of current research.

Over the past two decades, the *NAC* genes have been identified in many species, such as Arabidopsis (117) [5], maize (87) [32], rice (151) [33], millet (147) [34], soybean (152) [35], brachium (118) [36], durum wheat (168) [37], strawberry (112) [38] and ginseng (89) [39], and their structure, evolution, function and expression had been systematically analyzed. However, the functions and regulatory mechanisms of *NAC* family members in secondary metabolism have largely not been studied in ginseng. Therefore, in this study, based on the previous identification of the *PgNAC* gene family in the laboratory, the *PgNAC* gene was located on the ginseng chromosome, and the *NAC* genome replication phenomenon was found to also occur in ginseng. We selected *NAC* genes highly related to ginsenoside synthesis, and systematically analyzed their structure, evolution and expression. By *Rhizobium rhizogenes* mediated genetic transformation, the role of a NAC transcription factor in ginsenoside synthesis was examined. This study should serve as a reference for the in-depth study of the ginseng NAC transcription factor family’s role in the molecular regulation mechanism of ginsenoside biosynthesis and for a future industrial production of ginsenoside.

## 2. Results

### 2.1. Chromosome Localization and Gene Replication of the PgNAC Gene

**Figure 1:**
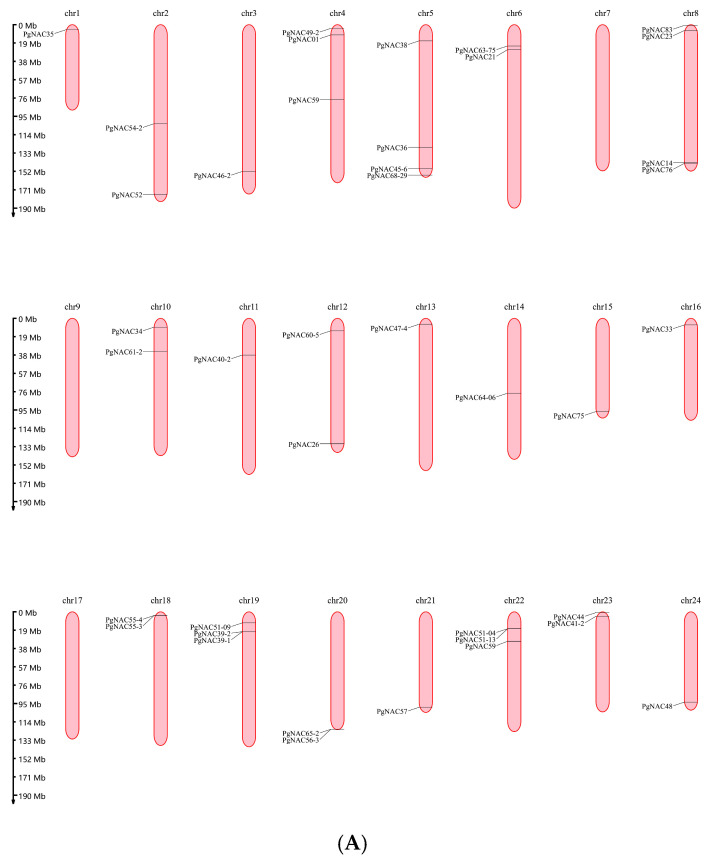

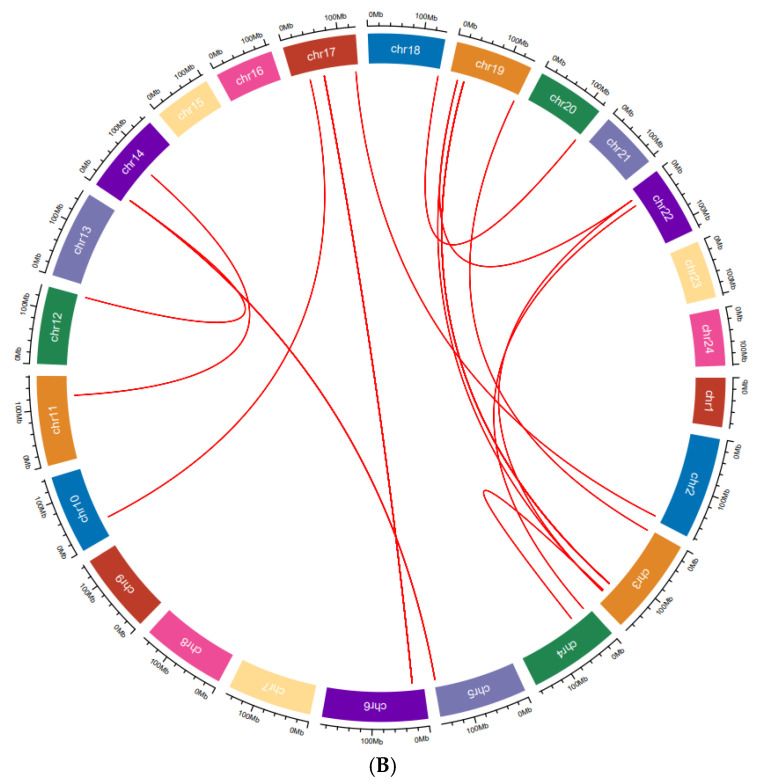
Chromosome localization and covariance analysis of the *PgNACs* genes. (**A**) The physical locations of *NAC* genes on ginseng chromosomes; the scale is in megabases, Mb. (**B**) The Circos plot shows the relative positional relationship of the *NAC* genes in ginseng, where each colored band represents a chromosome of ginseng; the ends of the red lines point toward paralog pairs derived from segmental duplication.

Among the identified *PgNAC* genes, 77 *PgNAC* genes were located on 24 pairs of chromosomes of ginseng. No *NAC* members were identified on chromosomes 7, 9, and 17; chromosome 5 contained the largest number of *PgNAC* members (27) (Figure 1A). In conclusion, *PgNAC* gene family members are not evenly distributed on ginseng chromosomes. There are very complex collinearity relationships among *PgNAC* gene family members, due to multiple *PgNAC* gene replication events in ginseng (Figure 1B).

### 2.2. Identification of PgNAC Genes Highly Associated with Ginsenoside Synthesis

In order to study the relationship between *PgNAC* genes and ginsenoside biosynthesis, the Pearson correlation coefficient in SPSS Version 23.0 software was used to analyze the correlation between the expression level of *PgNACs* and the content of saponins (nine monomeric saponins and total saponins). The results (Table S1) shown that a total of 94 *PgNAC* genes were correlated with the content of saponins. There were significant (*p* ≤ 0.05) or highly significant (*p* ≤ 0.01) correlations with the content of one or more monomeric saponins and of total saponins. A total of 17 genes were significantly correlated with total saponins, and 5 genes with total saponins. The Pearson correlation coefficient approach with SPSS Version 23.0 software was used to analyze the correlation between the expression of *PgNACs* and that of key enzyme genes. The results (Table S2) showed that 122 genes were significantly correlated with the expression of key enzyme genes. The genes related to both saponin content and the expression of key enzyme genes were sorted out, the genes with incomplete conserved domains or open reading frames were removed and 24 *PgNACs* were obtained (*PgNAC41-2*, *PgNAC44*, *PgNAC47-2*, *PgNAC51-09*, *PgNAC54-1*, *PgNAC54-2*, *PgNAC56-1*, *PgNAC59*, *PgNAC60-3*, *PgNAC60-5*, *PgNAC61-2*, *PgNAC65-9*, *PgNAC66-16*, *PgNAC66-02*, *PgNAC66-22*, *PgNAC66-31*, *PgNAC66-04*, *PgNAC66-08*, *PgNAC68-01*, *PgNAC68-15*, *PgNAC68-17*, *PgNAC68-19*, *PgNAC68-02* and *PgNAC68-06*).

**Figure 2:**
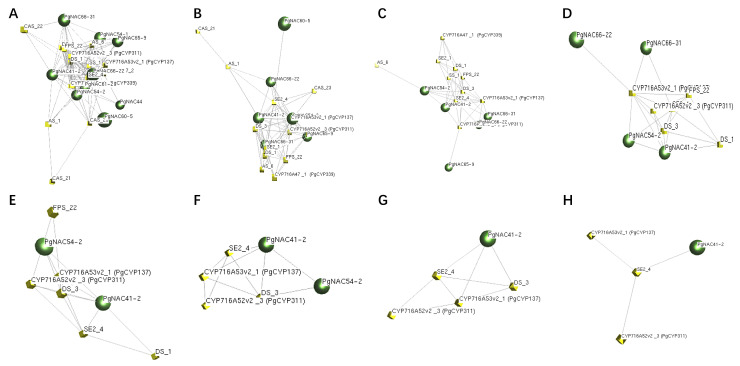
Interaction network between *PgNACs* genes and genes of key enzymes in ginsenoside synthesis. (**A**–**H**) When *p* value = 5.00 × 10^−2^, *p* value = 1.00 × 10^−2^, *p* value = 1.00 × 10^−3^, *p* value = 1.00 × 10^−4^, *p* value = 1.00 × 10^−5^, *p* value = 1.00 × 10^−6^, *p* value = 1.00 × 10^−7^ and *p* value = 1.00 × 10^−8^. This result shown the interaction network between *PgNAC* genes and ginsenoside synthesis key enzyme genes. Green represents the *PgNAC* genes, and yellow represents the key enzyme genes of ginsenoside synthesis.

The 24 genes obtained and 16 key enzyme genes verified were analyzed by the interaction network under different *p* values (Figure 2). When *p* value = 5.00 × 10^−2^ to *p* value = 1.00 × 10^−8^, only the gene *PgNAC41-2* was found always clustered with the key enzyme genes, with a tight connection. Also, *PgNAC41-2* was significantly correlated with the contents of Re, Rb2 and total saponins, and significantly correlated with the contents of Rb1 and Rb3, too. These results indicated that the *PgNAC41-2* gene quite likely plays a role in regulation of key enzyme genes in the saponin synthesis pathway. Thus, the *PgNAC41-2* gene was selected for the further functional verification.

### 2.3. Sequence Analysis of the PgNAC41-2 Gene

**Figure 3:**
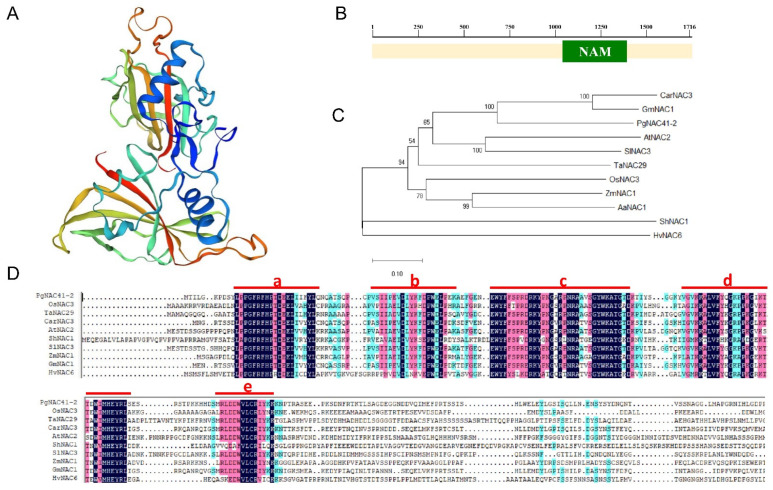
Sequence analysis of the *PgNAC41-2* gene. (**A**) Tertiary structure of the derived PgNAC41-2 protein. (**B**) Conserved domain of the PgNAC41-2 protein. (**C**) Evolutionary analysis of *NAC* genes in different species. (**D**) Multiple amino acid sequence alignment derived from PgNAC41-2: rice OsNAC3, wheat TaNAC29, chickpea CarNAC3, Arabidopsis AtNAC2, tomato ShNAC1, tomato SlNAC3, corn ZmNAC1, soybean GmNAC1 and barley HvNAC6. The NAC domain with five conserved regions (a–e) are indicated by red lines.

The ORF of *PgNAC41-2* is 861 bases long. The protein encoded consists of 286 amino acids with a molecular mass of 32,686.66 kDa and an isoelectric point of 6.61. The secondary structure comprises 61 α-helices, accounting for 21.33%; there were 12 β-folds, accounting for 4.20%; and there were 173 irregular curls, accounting for 60.49%. The tertiary structure is shown in Figure 3A. In general, there is a conserved domain within the NAM isoform in NAC, called the NAM domain. Conserved domain analysis revealed that PgNAC41-2 contained a NAM domain that was 1039–1413 in length (Figure 3B). To reveal the evolutionary relationships between NAC, phylogenetic trees were constructed using the protein sequences derived from PgNAC41-2 and *NAC* gene family members of nine other species (rice OsNAC3, wheat TaNAC29, chickpea CarNAC3, Arabidopsis AtNAC2, hairy tomato ShNAC1, tomato SlNAC3, corn ZmNAC1, soybean GmNAC1 and barley HvNAC6) (Table S3). Figure 3C shows the evolutionary relationships of the 10 NAC genes. PgNAC41-2 has the closest evolutionary distance with soybean GmNAC1 and chickpea CarNAC3. Figure 3D shows 10 NAC protein sequences with five conserved domains (Figure 3D: a–e) in the NAC domain.

### 2.4. Expression Pattern Analysis of the PgNAC41-2 Gene

**Figure 4:**
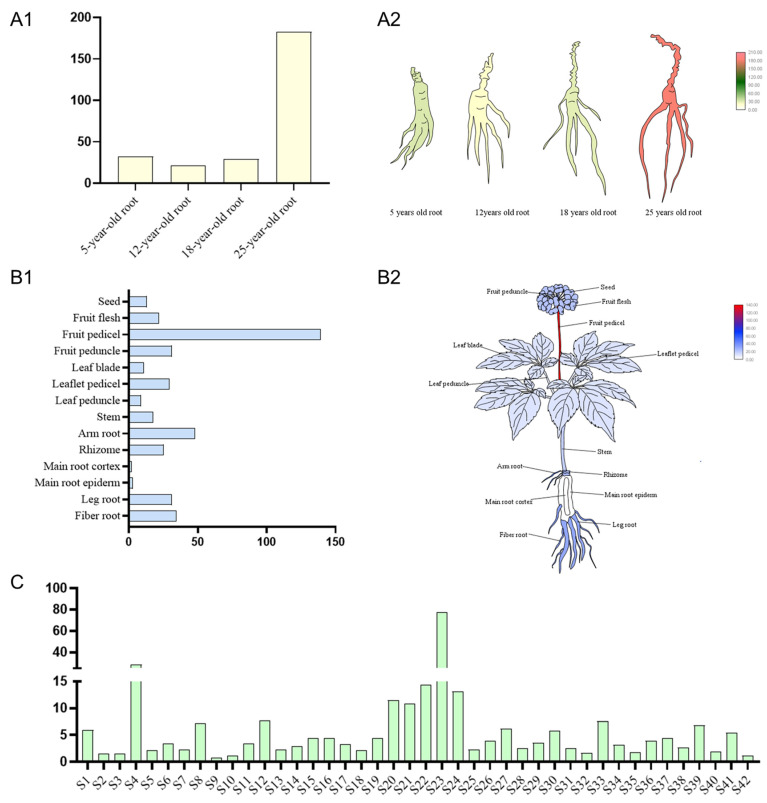
Expression analysis of the *PgNAC41-2* gene. (**A1**) Expression of the *PgNAC41-2* gene in four ginseng roots of different ages. The X axis represents ginseng of different ages, and the Y axis represents TPM values of gene expression. (**A2**) Heat map of the *PgNAC41-2* gene expression in four ginseng roots of different ages, with yellow to green and then to orange indicating, in turn, that the expression increased. (**B1**) Expression of the *PgNAC41-2* gene in 14 different tissues. The X axis represents the TPM values of gene expression, and the Y axis represents the different tissues of ginseng. (**B2**) From white to blue and then to red, indicating that the expression of the *PgNAC41-2* gene increased successively in 14 different tissues. (**C**) Expression of the *PgNAC41-2* gene in 42 farm cultivars. The X axis represents the different farm cultivars of ginseng, and the Y axis represents the TPM values of gene expression.

In order to further investigate the expression pattern of the *PgNAC41-2* gene in ginseng, we retrieved the expression data of *PgNAC41-2* gene from 14 different tissues, four different age stages (5, 12, 18 and 25 years) and 42 farmers’ cultivars of 4-year-old ginseng roots contained in database 1, and constructed heat maps. As shown in Figure 4A1,A2, the expression of the *PgNAC41-2* gene was the highest in 25-year-old ginseng roots, and the lowest was in 12-year-old ginseng roots. The expression of *PgNAC41-2* in 14 different tissues (Figure 4B1,B2) showed that it was expressed in all tissues, with the highest expression level in the fruit stem, a higher expression level in fibrous root and leg root and the lowest expression level in the primary root cortex and the rhizome. Among the 42 farmers’ cultivars (Figure 4C), the expression of the *PgNAC41-2* gene was similar in most of the farm varieties, and the expression of the *PgNAC41-2* gene was high only in S4 and S23.

### 2.5. PgNAC41-2 Gene Cloning and Vector Construction

**Figure 5:**
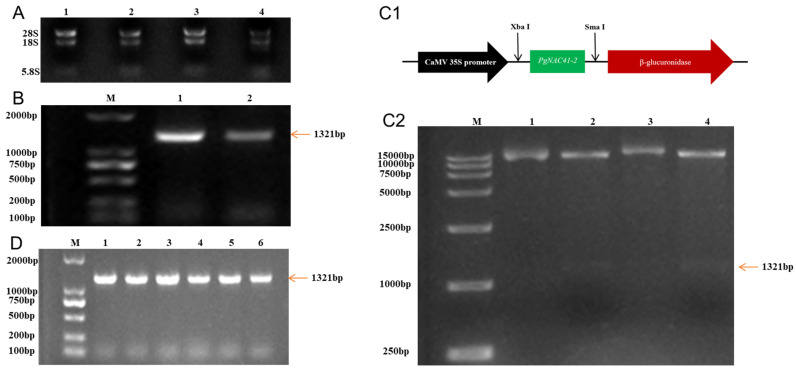
Cloning of the *PgNAC41-2* gene and construction of the vector. (**A**) RNA electrophoretic pattern. (**B**) PCR electrophoretic diagram of the *PgNAC41-2* gene transferred into the competent DH5α. (**C1**) Overexpression vector diagram of PBI121-PgNAC-41-2 constructed. (**C2**) Double digestion electrophoresis of recombinant plasmids. (**D**) Transfer of recombinant plasmids into genetically engineered bacteria.

The results of ginseng total RNA agarose gel electrophoresis are shown in Figure 5A; 18S and 28S are clearly seen. The reverse transcription reaction of RNA obtained in the previous step was carried out with a reverse transcription kit, and cDNA was successfully obtained. The *PGNAC41-2* gene fragment was amplified by PCR using cDNA as template, and the target fragment length was 1321 bp.

The successfully constructed cloning vector was transferred into competent *Escherichia coli* cells for blue and white detection. After overnight culture, white colonies were selected into liquid LB medium, and the PCR results of bacterial liquid are shown in Figure 5B. The *E. coli* plasmid and vector plasmid pBI121 were double digested; the target gene was recovered after *E.coli* plasmid digestion, and the plasmid pBI121 was double-digested for recovery. The target gene after double digestion was ligated with T4 ligase to construct the expression vector (Figure 5C1), and then the expression vector was transferred into Escherichia coli. Single colonies were selected for resistance screening, and the positive clones were verified by double digestion. Figure 5C2 shows that the expression vector was successfully constructed.

### 2.6. Genetic Transformation with the PgNAC41-2 Gene

**Figure 6:**
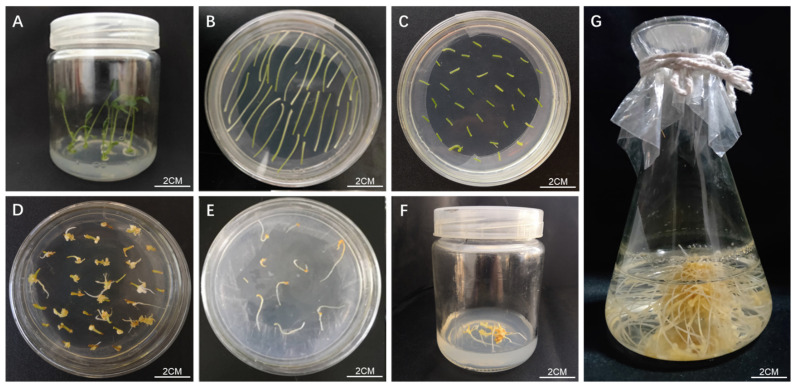
Hairy roots and propagation induced by *PgNAC41-2* gene transformation. (**A**) Ginseng sterile vaccine. (**B**) Preculture of petiole of sterile seedlings. (**C**) Co-culture. (**D**,**E**) Hairy roots infected by *Rhizobium rhizogenes*. (**F**) Solid culture of hairy roots. (**G**) Liquid culture of hairy roots. ((**A**,**B**) under light; (**C**–**G**) placed in dark culture).

The constructed overexpression vector was transformed into competent *Rhizobium rhizogenes* A4 cells to prepare genetically engineered bacteria. Figure 5D shows that the expression vector was successfully transferred into engineered bacteria. After infection with *Rhizobium rhizogenes* A4, ginseng explants were cultured under dark conditions (Figure 6A,B). After 4 weeks, the originating roots began to grow on the explants (Figure 6C,D). When the hairlike roots grew to 3–4 cm long (Figure 6E,F), the hairlike roots were transferred to a new medium for propagation (Figure 6G).

**Figure 7:**
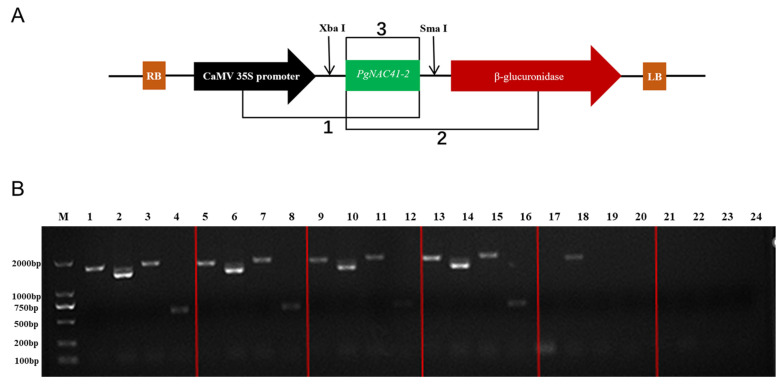
Three-fragment PCR analysis of ginseng hairy roots transformed with *PgNAC41-2* gene. (**A**) Three fragments cloned by the three-segment PCR method. (**B**) M: marker; 1–16: positive plants; 17–20: negative control; 21–24: blank control.

Three-segment PCR was used to screen positive ginseng hairlike root clones containing the *PgNAC41-2* gene, as shown in Figure 7A, to design primers 1, 2 and 3. The genomic DNA of hair roots was extracted by TPS method; the wild-type hair roots were used as a negative control, and water was used as a blank control for preliminary PCR detection. The results are shown in Figure 7B. The length of the band was correct, bright and clear, and the *PgNAC41-2* gene was initially confirmed to be transferred into the hair roots.

### 2.7. Functional Verification of PgNAC41-2 Gene

**Figure 8:**
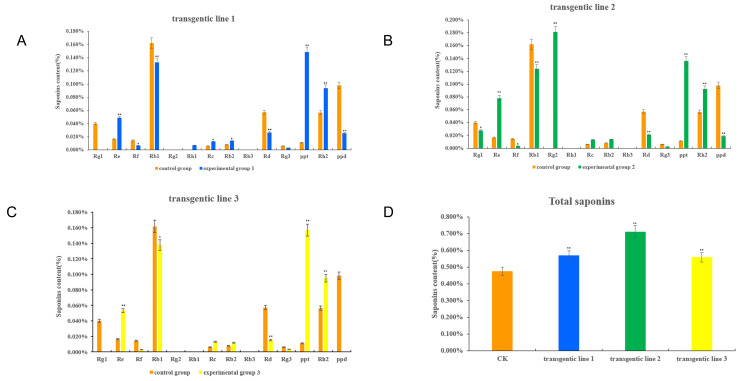
Determination of ginsenoside content. (**A**) Transgenic line 1. The abscissa represents the different saponin types, and the ordinate represents the saponin content (µg/mg). (**B**) Transgenic line 2. The abscissa represents the different saponin types, and the ordinate represents the saponin content (µg/mg). (**C**) Transgenic line 3. The abscissa represents the different saponin types, and the ordinate represents the saponin content (µg/mg). (**D**) Total saponins. The abscissa represents the different hair roots and the ordinate represents the saponin content (µg/mg). *. At the 0.05 level (two-tailed), the association was significant. **. At the 0.01 level (two-tailed), the association was significant.

Three positive hairlike roots were selected, and the content of monomeric saponins in the positive hairlike roots was detected by High Performance Liquid Chromatography (HPLC). The detection results are shown in Figure 8. Compared with the content of monomeric saponins in the wild-type hairlike roots, the contents of Re, Rh2 and aglycone PPT in the single root of the three positive hairlike roots were extremely significantly increased. Rc was significantly increased in the single root of positive hair root 1, and extremely significantly increased in the single root of positive hair root 2 and the single root of positive hair root 3. Rb2 was significantly increased in positive hair root single root 1 and positive hair root single root 3, and extremely significantly increased in positive hair root single root 2. At the same time, the content of total saponins in the single root of the three positive hair roots changed significantly, indicating that the *PgNAC42-1* gene promoted saponin biosynthesis.

## 3. Discussion

*Panax ginseng* is a tetraploid plant with 24 pairs of chromosomes [40]. Most members of the *PgNAC* gene family are distributed on *Panax ginseng* chromosomes, and only a few chromosomes do not have *NAC* transcription factors genes. Tandem replication events are important events that drive the function of new organisms. Gene replication of the *NAC* gene family also exists in ginseng, and in *Eucommia ulmoides* [41], *Saccharum spontaneum* [42] and *Kandelia obovate* [43] the same phenomenon of gene duplication is observed. When the *NAC* transcription factor *AaNAC1* in *Artemisia annua* was overexpressed, the content of artemisinin and dihydroartemisinic acid increased, and the expression level of artemisinin biosynthesis pathway genes also increased [18]. Rice transcription factor *OsNAC6* can positively regulate the biosynthesis of nicotinamide in roots [19]. In papaya, transcription factor *CpNAC1* plays a positive regulatory role in carotenoid biosynthesis by activating the expression of *CP-PDS2/4* [22]. Based on the correlation analysis between the expression level of *PgNACs* and saponin content as well as the expression level of key enzyme genes for ginsenoside synthesis, only a subset of *PgNAC* genes were found to be significantly correlated with both saponin content and the expression level of the verified key enzyme genes, further demonstrating the functional diversity of *NAC* gene family members. It was also found that the *PgNAC41-2* gene was significantly correlated with the saponin content and the expression of key saponin synthesis enzyme genes. The *PgNAC41-2* gene was significantly correlated with monomeric saponins Re, Rb2 and total saponins, and was significantly correlated with monomeric saponins Rb1 and Rb3. The *PgNAC41-2* gene was significantly correlated with key enzyme genes *CYP716A52v2_1*, *CYP716A52v2_3*, *CYP716A47_1*, *DS_1*, *DS_3*, *β-AS_1*, *SS_1*, *SE2_4*, *FPS_22* and *UGT71A27_2*. And the key enzymes genes *β-AS_6*, *CAS_22*, *CAS_23* and *SE2_1* were significantly correlated. When the *p* value became strict, the network expression analysis of key enzyme genes revealed that the *PgNAC41-2* gene was always connected to the key enzyme gene *SE2_4*. This further suggested that *PgNAC41-2* expression is most closely related to that of key enzyme genes, which suggests a regulatory role in saponin synthesis. Therefore, we selected this gene for further study.

The main structural feature of the *NAC* transcription factor is its N-terminal part, which contains a highly conserved and specific NAC domain, which can bind DNA and other proteins, while the C-terminal part has a highly variable transcriptional regulatory region [44] that can activate or inhibit gene transcription and thereby participate in plant growth and development, hormone signal transduction and secondary metabolite synthesis. The evolutionary analysis showed that the PgNAC41-2 had the closest evolutionary distance to soybean GmNAC1 and chickpea CarNAC3, and the farthest to barley HvNAC6. The amino acid alignment revealed that the sequences near the N-terminus were similar due to the highly conserved and specific NAC domain, while the C-terminus region was very variable, indicating that this region conferred different functions to NAC proteins through a selective interaction between *NAC* transcription factors and various target proteins.

Analysis of the expression pattern of *PgNAC41-2* yielded several interesting findings. Firstly, the expression level of *PgNAC41-2* was analyzed in 42 farmers’ cultivars, and it was expressed in all varieties, which indicated that the expression of *PgNAC41-2* was extensive. However, the *PgNAC41-2* was specifically expressed in farmers’ cultivars S4 and S23. Interestingly, the expression of the *PgNAC41-2* gene in 14 different tissues of four-year-old ginseng was also specific, and the highest expression level was found in the fruit stem. Furthermore, the expression of *PgNAC41-2* showed an obvious trend to specifically increase with time in 12-, 18-, and 25-year-old plants, being most prominent in roots of 25-day-old plants. Although *PgNAC41-2* has been identified by modern bioinformatics methods, the regulatory mechanism of *PgNAC41-2* gene involved in saponin synthesis is still unclear and needs to be further studied.

Dalman et al. [25] showed that the level of specific flavonoids in Norwegian spruce lines overexpressing *PaNAC03* decreased. *PdWND3A* in *Populus deltoides*, coding for a member of a protein family containing a NAC domain, regulates lignin biosynthesis by its effect on the expression of the *F5H* gene [24]. All these observations support the hypothesis that *NAC* transcription factors play an important regulatory role in plant secondary metabolite biosynthesis. Accordingly, we found that the contents of Re, Rc, Rb2, PPT, Rh2 and total saponins were increased in the positive hair roots of *PgNAC41-2* transgenic plants. The correlation analysis based on statistics can serve as a significant reference for screening genes involved in saponin synthesis, and also as a starting point for further studies. Clearly, the quantification of monomeric saponins showed that overexpression of the *PgNAC41-2* gene promoted their biosynthesis; *GRAS* transcription factor *PgGRAS68-01* [45] and *Trihelix* transcription factor *PgGT25-04* [46] are both involved in the biosynthesis of ginsenosides. All these suggest that transcription factors can be involved in the regulation of secondary metabolism in ginseng, but the underlying regulatory mechanism will need further investigation.

## 4. Materials and Methods

### 4.1. Plant Materials and Data Sources

The ginseng used in this experiment was from the Jilin Engineering Research Center Ginseng Genetic Resources Development and Utilization, and the transformed material was derived from sterile, germinated seeds from ginseng. All the bacteria and vectors were provided by the laboratory. The data used in this study were all from the transcriptome database of the Jilin Ginseng Center and constructed in the laboratory [47]. The present study was based on the *PgNAC* gene family identified in the transcriptome database of ginseng in Jilin [39]. The contents of ginsenosides (9 monomer saponin and total ginsenosides) and the verified expression levels of key enzyme genes related to ginsenoside synthesis were also obtained from the Jilin Ginseng Center, comprising 42 different regions.

### 4.2. PgNAC Gene Duplication and Chromosome Localization

We used Blastn to compare the identified *PgNAC* genes [39] with the ginseng genome to determine their distribution in the ginseng genome [48] and the replication of the *PgNAC* gene family in the ginseng genome. Identity ≥ 99%, coverage length ≥ 350 bp and *E*-value ≤ 1.0 × 10^−100^ were used as criteria for the comparison, the R-Package Circlize [49] structure of this gene was used to determine the gene replication phenomenon of this gene family in the ginseng genome. Identity = 100% (when the identity was found the same, the one with the longer length was chosen) and *E*-value ≤ 1.0 × 10^−100^ were used as criteria for the comparison; the location of the transcripts on the chromosomes was visualized using the MG2C online tool (http://mg2c.iask.in/mg2c_v2.1/in-dex.html, accessed on 1 June 2022).

### 4.3. Identification of PgNACs Genes Related to Ginsenoside Synthesis

From the database [39], the relative expression levels of *PgNACs* in the roots of 42 farm varieties of four-year-old ginseng were obtained. The Pearson correlation coefficient in SPSS Version 23.0 software [50] was used to analyze the correlation between the relative expression of *PgNACs* and the content of saponins, as well as the correlation between the expression of *PgNACs* and the verified 16 key enzyme genes [45]. Network analysis was conducted between these genes and key enzyme genes. The co-expression networks were constructed using BioLayout Express^3D^ Version 3.2 Software [51]. Under the condition of a gradually strict *p* value, *PgNACs* with closer connection to key enzyme genes were screened to obtain candidate genes for the next experiment.

### 4.4. Sequence Analysis of PgNAC41-2

The online software Expasy ProtParam (https://web.expasy.org/protparam/, accessed on 12 June 2022) was used to predict the PgNAC41-2 protein basic physical and chemical properties of protein, including the relative molecular mass (in kDa) and its isoelectric point (PI). With the aid of two online software packages, SOPMA (https://npsa-prabi.ibcp.fr/cgi-bin/npsa_automat.pl?page=/NPSA/npsa_sopma.html, accessed on 14 June 2022) [52] and SWISS-MODEL (https://swissmodel.expasy.org/, accessed on 14 June 2022) [53], respectively, we analyzed the secondary and tertiary structure of the PgNAC41-2 amino acid sequence. NCBI ORF Finder software (http://www.ncbi.nlm.nih.gov/orffinder/, accessed on 14 June 2022) was used to find a complete conservative *PgNAC41-2* gene structure domain. To construct the phylogenetic tree from the protein sequences derived from PgNAC41-2 and from 9 other species, the neighbor-joining method of MEGA-X was used, and the boot repeat was set to 2000. These protein sequences were aligned using DNAMAN Version 5.2.2 software, with parameters set to default values.

### 4.5. Expression Pattern Analysis of PgNAC41-2

In order to further analyze the expression pattern of *PgNAC41-2* in ginseng, we selected the expression levels of *PgNAC41-2* in 14 different tissues, 4 different age stages (5, 12, 18 and 25 years) and 42 farmers’ cultivars of 4-year-old ginseng roots, using the database [39] and TBtools Version 1.0987 software [54] to visualize the expression levels.

### 4.6. Cloning of PgNAC41-2 Gene and Vector Construction

Total RNA from ginseng was extracted by the TRIZOL method, its concentration and integrity were determined, and reverse-transcribed into cDNA using the SPARKscript II RT Plus kit (With gDNA Eraser) (Shandong Sparkjade Biotechnology Co., Ltd., Qingdao, China). Specific primers were designed in the upstream and downstream of the ORF region of *PgNAC41-2* gene. The 5′ end of the upstream primer was added with *Xba*I restriction recognition sequence and its protective base, and the 5′ end of the downstream primer was added with *Sma*I restriction recognition sequence and its protective base. Forward primer: 5′-TCCCCCGGGCAAACACTCTCTCTTCTCTCCTCTC-3′, the reverse primer: 5′-TGCTCTAGATTGACTTGGGCATCCATCTG-3′. Using the Touchdown PCR amplification system, the first cycle number was 20 cycles, each cycle was reduced by 0.5 °C, and the second cycle number was 15 cycles. After amplification, *PgNAC41-2* was obtained. The cloned DNA fragments were then sent for sequencing by Sangon Biotech (Shanghai, China).

The amplified product fragments were inserted into pMD™ 18-T Vector Cloning Kit (Sangon Biotech Co., Ltd., Shanghai, China), then transferred into competent *E. coli* cells DH5α for culture and identified by sequencing. After correct identification, the candidate *PgNAC41-2* gene fragment was constructed into the pBI121 expression vector and verified by *Xba*I and *Sma*I double digestion. The candidate *PgNAC41-2* gene overexpression vector PBI121-PgNAC41-2 was successfully constructed.

### 4.7. Genetic Transformation of Ginseng Explants

To prepare genetically engineered bacteria, the PBI121-PgNAC41-2 overexpression vector was constructed to transform competent *Rhizobium rhizogenes* A4 cells. The petioles of ginseng sterile seedlings were cut into 1 cm long segments and put into MS solid culture dishes. The seedlings were precultured for 3 days at 22 °C with photoperiod and subsequently used. The recombinant plasmid pBI121-PgNAC41-2 was transformed into *Rhizobium rhizogenes* A4 for infecting the petioles of ginseng sterile seedlings. The ginseng hair roots with cephalosporin resistance (Cef) were screened on MS solid medium. After the resistant hairy roots had grown to 1–2 cm, they were inoculated in new 1/2MS medium for culture. Thereafter, the positive ginseng hair root clones containing the candidate *PgNAC41-2* gene were screened by three-segment PCR (The three-fragment PCR method is to amplify three target fragments. The first amplified fragment contained the target gene and the vector fragment upstream of the target gene. The second amplified fragment included the target gene and the target gene downstream vector fragment. The third amplified fragment was the target gene). The positive hairy roots were then propagated once a month until they were propagated to the amount required to measure saponin content. Ginseng hair roots were cultured at 24 °C in the dark.

### 4.8. Functional Verification of PgNAC41-2 Gene

The positive hair roots with a dry weight of 1 g were weighed and ground into powder. The cold-treated hair roots with a dry weight of 1 g were weighed and ground into powder, wrapped with filter paper, soaked in 30 mL of 80% methanol overnight, extracted by ultrasound for 30 min, then added to 70 mL of 80% methanol solution, put into Soxhlet extractor, and extracted by 90% for 30 h to collect methanol solution. The samples were dried using a rotary evaporator, redissolved in 10 mL of 100% methanol solution, and filtered through 0.22 μm microporous filter membrane for reserve use. Ginsenoside was measured with the use of a high-performance liquid chromatograph (Waters e2695 Separations Module, Waters Listed Company, Milford, MA, USA). The chromatographic conditions for the detection of ginsenoside were as follows: Waters C18 column (4.6 mm × 250 mm, 5 μm); mobile phase: A. acetonitrile, B. water, using gradient elution method, as shown in Table 1, A, 18%, B, 82%, mobile phase flow rate 1.0 mL/min, column temperature 35 °C, injection volume 20 μL, detection wavelength 203 nm.

Preparation of ginsenoside standard solution: ginseng monomeric ginsenoside standard Rg1, Re, Rf, Rb1, Rg2, Rh1, Rc, Rb2, Rb3, Rd, Rg3, ppt, Rh2, ppd were accurately weighed, and methanol was added. The standard solution was filtered by 0.22 μm filter membrane for later use.

## 5. Conclusions

In this study, a lot of *PgNAC* genes were located on 24 pairs of chromosomes of ginseng, and *PgNAC* gene replication was found in ginseng. The correlation analysis between the expression level of *PgNACs* and the content of saponins and the expression levels of key enzyme genes showed that 94 *PgNACs* were correlated with the content of saponins and that 122 *PgNACs* were significantly correlated with the expression levels of key enzyme genes. There were 60 genes significantly correlated with both the content of saponins and the expression of key enzyme genes, indicating that *PgNACs* were directly or indirectly involved in the regulation of ginsenoside synthesis. The candidate gene *PgNAC41-2* was identified by the interaction network analysis between *PgNAC* genes and key enzyme genes. The analysis of the sequence, phylogeny and expression pattern of the *PgNAC41-2* gene revealed its specific temporal and spatial expression. Subsequently, we cloned this *PgNAC41-2* gene, constructed the plant expression vector, and transformed ginseng explants by a well-established *Rhizobium rhizogenes* method. After PCR preliminary identification, positive hairy roots were obtained. The contents of Re, Rc, Rb2, PPT, Rh2 and total saponins in positive hair roots were significantly increased, indicating that the *PgNAC42-1* gene promoted the biosynthesis of saponins.

## Figures and Tables

**Table 1:** Mobile phase gradient condition table for HPLC.

Time (min)	Solvent A (%)	Solvent B (%)	Flow Velocity (mL/min)
0~40	18~21	82~79	1
40~42	21~26	79~74	1
42~46	26~32	74~68	1
46~66	32~33.5	68~66.5	1
66~71	33.5~38	66.5~62	1
71~86	38~65	62~35	1
86~91	65	35	1
91~96	65~85	35~15	1
96~103	85	15	1
103~105	85~18	15~82	1
105~106	18	82	1

## Data Availability

All ginseng samples and ginseng hairy roots materials were stored in Jilin Agricultural University and Jilin Engineering Research Center Ginseng Genetic Resources Development and Utilization. All ginseng data used for this study are available at National Center for Biotechnology Information (NCBI) under BioProject PRJNA302556. All ginseng materials are available through corresponding authors upon request.

## Supplementary Materials

The following supporting information can be downloaded at: https://www.mdpi.com/article/10.3390/ijms241511946/s1.
